# Chemical composition and roughness of enamel and composite after bleaching, acidic beverages and toothbrushing

**DOI:** 10.4317/jced.56442

**Published:** 2019-12-01

**Authors:** Marília-de-Morais Pinelli, Anderson Catelan, Luís-Felipe-Marques de Resende, Luís-Eduardo-Silva Soares, Flávio-Henrique-Baggio Aguiar, Priscila-Christiane-Suzy Liporoni

**Affiliations:** 1 Graduate Student, Department of Dentistry, University of Taubaté, Taubaté, SP, Brazil; 2 Assistant Professor, Department of Dentistry, University of Taubaté, Taubaté, SP, Brazil; 3 Assistant Professor, Graduate Program in Dentistry, Faculty of Health Sciences, University of Western São Paulo, Presidente Prudente, SP, Brazil; 4 Assistant Professor, Laboratory of Dentistry and Applied Materials (LDAM), Research and Development Institute (IP&D), University of Vale do Paraíba, São José dos Campos, SP, Brazil; 5 Associate Professor, Department of Restorative Dentistry, Piracicaba Dental School, University of Campinas, Piracicaba, SP, Brazil

## Abstract

**Background:**

In this study was assessed the surface roughness and chemical composition of tooth enamel and composite resin after bleaching treatment, immersion in acidic beverages, and simulated toothbrushing.

**Material and Methods:**

One hundred and twenty dental blocks (10 x 10 x 3 mm) were randomly assigned (n = 10) according to surface treatment [none (N), bleaching (B), toothbrushing (T), and B+T] and storage medium [saliva (S), whiskey (W), and orange juice (O)]: experimental groups - N+S, N+W, N+O, B+S, B+W, B+O, S+T, W+T, O+T, B+S+T, B+W+T, and B+O+T. Two bleaching sessions were conducted using 38% hydrogen peroxide (3 applications). Surface roughness was measured using a roughness tester and composition was determined by micro energy-dispersive X-ray fluorescence spectrometry (µ-EDXRF) before and after treatments. Calcium/phosphorus (Ca/P) ratio in enamel and silica (Si) content in composite were evaluated. Data were statistically analyzed by ANOVA and Tukey’s test (α = 0.05).

**Results:**

Overall, increased values of surface roughness for enamel and composite were observed mainly after immersion in orange juice and bleaching/toothbrushing association. Moreover, this association and immersion in whiskey resulted in lower Ca/P ratio and after aging methods, bleached and bleached/toothbrushed groups showed decreased in Ca/P ratio compared to initial values. All groups showed Si content decrease at the end, except the group without surface treatment and immersed in saliva, and bleaching followed by immersion in orange juice and toothbrushing caused the highest Si reduction.

**Conclusions:**

Bleaching and toothbrushing combination strengthened the effects caused by acidic drinks on roughness and chemical composition of enamel and composite.

** Key words:**Tooth bleaching, toothbrushing, physical properties, chemical properties.

## Introduction

Smile esthetics is an important factor in individual presentation to social acceptance. Thus, dental bleaching is a conservative technique, which allows that natural or stained teeth to be changed without wearing the tooth structure, improving esthetic appearance of teeth (1). On the other hand, indiscriminate use of bleaching agents can increase the porosity and erosion on enamel surface, even using low concentration products (2,3).

Hydrogen peroxide (HP) at high concentration constitutes an alternative in cases of severe color alteration when patients have difficulty using a tray or fast treatment is needed. However, this bleaching agent causes on enamel surface increased roughness, alterations in inorganic composition and organic matrix, and decreased hardness (1,4-9).

Mineral loss and erosion of tooth hard structure can be intensified by consumption of acidic food and drinks (10,11). So, the interaction of bleaching agents with intake of acidic drinks could intensify the damage on enamel surface (10,12). Likewise, increased surface roughness of resinous materials has been reported after bleaching procedure (13,14) and intake of low pH drinks (15). In addition, abrasion caused by toothbrushing also increases the roughness (16,17).

In order to assess alterations in mineral content of enamel and inorganic component of composite, a sensitive chemical analysis can be performed using energy dispersive X-ray microanalysis (3,12). This analysis is a non-destructive method, which provides information about atomic and structural composition of substrate (12).

Thus, the purpose in this study was to evaluate the surface roughness and change in chemical composition of tooth enamel and composite resin submitted to bleaching treatment using 38% HP, immersion in low pH solutions, and/or simulated toothbrushing. The research hypothesis was that roughness and chemical composition of enamel and composite would be affected by the treatments.

## Material and Methods

-Specimen preparation

The 120 bovine incisors were disinfected in 0.1% thymol solution (Byofórmula Imp Exp, São José dos Campos, SP, Brazil) for 24 h at room temperature. Then, teeth were cleaned and stored in artificial saliva (Byofórmula). Saliva was changed every two days in order to maintain a standard in mineral conditions of enamel before initial analysis of roughness and chemical composition.

The roots were separated from dental crown using a precision saw (Isomet 1000; Buehler Inc., Lake Bluff, IL, USA). So, 120 square dental blocks (10 mm x 10 mm x 3 mm) were obtained from crown, buccal surfaces were polished with -600, -800, -1000, and -1200 grit silicon carbide abrasive papers (Buehler Inc.) using polishing machine (APL-4; Arotec, Cotia, SP, Brazil) under water irrigation.

In half of dental block, the enamel was left intact while in other half a cavity was prepared with high speed using a diamond bur wheel #3053 (KG Sorensen, Barueri, SP, Brazil). Cavity was restored with nanofilled composite resin (Filtek Z350, A3 shade, batch #BT5009; 3M ESPE, Saint Paul, MN, USA) and light cured for 20 s using a halogen unit (Soft-Start; Degussa Hüls, Postfach, Hanau, Germany) at 600 mW/cm2 of irradiance. Specimens were stored in distilled buffered water (Byofórmula) for 24 h at 37ºC. Then, restorations were polished with fine and superfine aluminum oxide abrasive discs (Sof-Lex Pop-On; 3M ESPE) for 15 s each disc.

After polishing procedure, specimens were stored in distilled buffered water for more 24 h at 37ºC. Afterwards, circular areas with 3 mm of diameter on enamel and composite were labeled with tape (Durex, 3M do Brasil Ltda, Sumaré, SP, Brazil), while the rest of surfaces were isolated with nail polish (Revlon Consumer Products Corp., Miami, FL, USA). After nail polish drying the tape was removed exposing the areas to be treated.

Specimens were randomly assigned in 12 groups (n = 10) according to surface treatment in 4 levels [none (N), bleaching (B), toothbrushing (T), and B+T] and storage medium in 3 levels [saliva (S), whiskey (W), and orange juice (O)]. Experimental design was as follows: group 1 (N+S), group 2 (N+W), group 3 (N+O), group 4 (B+S), group 5 (B+W), group 6 (B+O), group 7 (S+T), group 8 (W+T), group 9 (O+T), group 10 (B+S+T), group 11 (B+W+T), and group 12 (B+O+T). Groups were evaluated before (baseline) and after aging methods.

-Surface roughness

Arithmetical mean of roughness (Ra) was assessed using a profilometer (Surftest SJ-301; Mitutoyo Ltda., Tokyo, Japan) at constant speed of 0.5 mm/s and cut-off of 0.25 mm. Three readings in different positions were carried out with needle passing by center of specimen and rotating the same in approximately 120º after each measurement. Average of three readings was used as Ra for each specimen (14).

-Chemical composition

The chemical analysis was performed using a micro-energy dispersive X-ray fluorescence (µ-EDXRF) spectrometer (μEDX-1300; Shimadzu, Kyoto, Japan). Specimen surface was irradiated with X-ray beam of 50 µm and measurements were carried out in three points in enamel and in one point in composite. A semiconductor Si (Li) detector cooled by liquid nitrogen was used to radiation count. The tension and tube current were adjusted in 15 kV for enamel and 40 kV for composite, with reading time per point of 100 s and 25% dead time (12).

Stoichiometric synthetic hydroxyapatite (Sigma-Aldrich, St. Louis, MO, USA) was used as reference to enamel calibration with purity degree of 99.99% [Ca10(PO4)6(OH)2, batch #10818HA]. The variables to calculate the chemical formula were established for relative weights of calcium (Ca) and phosphorus (P) and the element oxygen was used as a chemical balance. The Ca/P ratio in enamel and silica (Si) content in composite were calculated by their relative weights as determined by direct reading of equipment.

-Bleaching procedure

Two bleaching sessions were performed using 38% HP (Opalescence Xtra Boost; Ultradent, South Jordan, UT, USA) with 3 applications in each session. In each application, 1 mL of bleaching agent was applied on specimen surface for 15 min. Then, gel was removed with gauze and a new application was done until completing 3 applications. Specimens were cleaned and stored in artificial saliva at 37ºC during a 7-day interval between the sessions.

-Solution immersion

Specimens were immersed in 10 mL of saliva (pH 7.1; Byofórmula), whiskey (Red Label, pH 3.7; Johnnie Walker, Kilmarnock, AD, Scotland), or orange juice (pH 3.6; Del Valle, Americana, SP, Brazil) for 10 min daily at 23ºC during 7 days. Afterwards, specimens were immersed in artificial saliva until completing a 24-h cycle. Solutions were changed daily. The pH of solutions was measured using a digital pH meter.

-Simulated toothbrushing

Specimens were submitted to 30,000 cycles of toothbrushing (Equilabor, Piracicaba, SP, Brazil), in line course of 20 mm extension, at a frequency of 4.5 cycles/s and constant load of 200 g. Oral B Indicator (Procter & Gamble, Manaus, AM, Brazil) medium-sized toothbrushes with soft-rounded bristles and a slurry (1:3) of whitening dentifrice (Colgate Ultra Branco; Colgate Palmolive, Osasco, SP, Brazil) and distilled water were used (12). At end, specimens were rinsed with running water and cleaned using an ultrasonic cleaner (USC-700; Unique, Indaiatuba, SP, Brazil) for 10 min.

-Statistical analysis

After exploratory data analysis, roughness, Ca/P ratio, and Si content data were statistically analyzed by three-way proc-mixed analysis of variance (ANOVA) for repeated measures followed by Tukey’s test at a pre-set of 5% significance level (version 9.1; SAS Institute Inc., Cary, NC, USA).

## Results

-Surface roughness

ANOVA showed interaction between the factors “surface treatment”, “storage medium”, and “time” for enamel and composite (*p* = 0.0001 and *p* = 0.0003, respectively).

In  Table 1, enamel bleached and toothbrushed presented the highest roughness values, followed by groups only bleached, which showed higher roughness compared to groups only toothbrushed. Bleached group immersed in whiskey showed the lowest roughness compared to group immersed in orange juice. In other surface treatments the results were statistically similar in both acidic drinks.

**Table 1:**
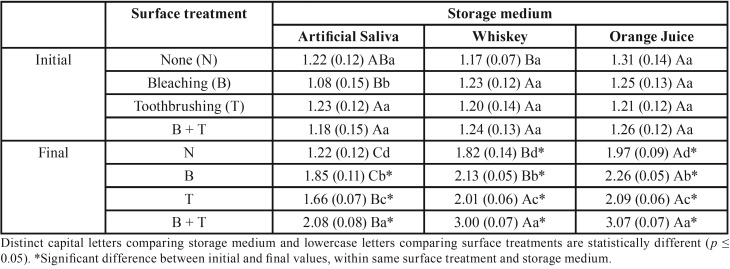
Surface roughness mean (standard deviation) of tooth enamel according to surface treatment and storage medium.

In  Table 2, bleaching and toothbrushing association also promoted higher surface roughness on composite, while the groups only bleached or toothbrushed showed similar roughness values. Group immersed in orange juice presented the highest roughness values compared to group immersed in whiskey, except for bleached group and immersed in whiskey.

**Table 2:**
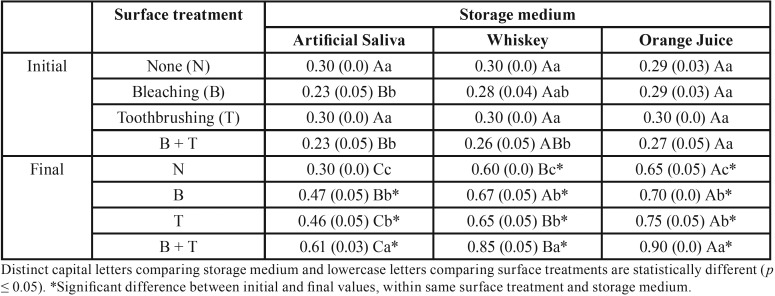
Surface roughness mean (standard deviation) of composite resin according to surface treatment and storage medium.

All groups showed an increase in values of surface roughness for enamel and composite, except for the group without surface treatment and immersed in artificial saliva (control). The lowest roughness was observed for group immersed in saliva, regardless of aging method.

-Chemical composition

ANOVA showed interaction between the factors “surface treatment”, “storage medium”, and “time” for enamel and composite (*p* = 0.0056 and *p* = 0.00001, respectively).

In  Table 3, bleached and toothbrushed groups showed similar Ca/P ratio, regardless of storage medium. Furthermore, bleaching/toothbrushing association and immersion in whiskey promoted lower Ca/P ratio compared to group immersed in saliva, while the group immersed in orange juice promoted intermediate values. After aging methods, bleached and bleached/toothbrushed groups showed decrease in Ca/P ratio, which was statistically significant when compared to baseline.

**Table 3:**
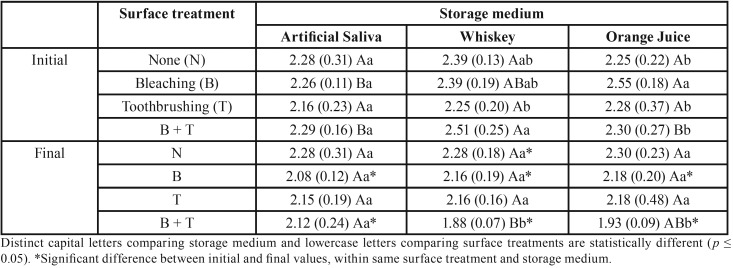
Calcium/phosphorus ratio mean (standard deviation) of tooth enamel according to surface treatment and storage medium.

In  Table 4, surface treatments and low pH solutions promoted higher change in Si proportion, but bleaching followed by immersion in orange juice and toothbrushing caused the highest reduction in Si content. Toothbrushed groups showed intermediate values without significant difference between acidic drinks. All groups showed Si decrease at the end, except the control group.

**Table 4:**
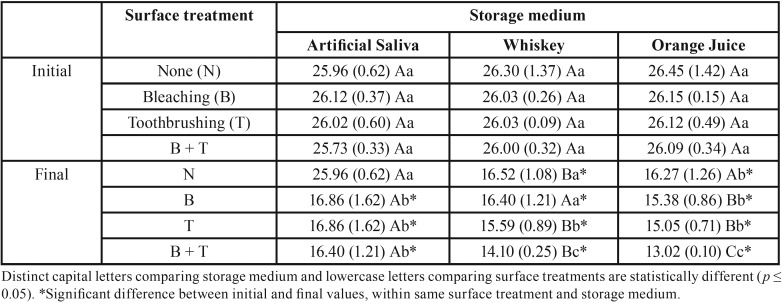
Silica content mean (standard deviation) of composite resin according to surface treatment and storage medium.

## Discussion

In the present study, bleaching treatment effects on enamel and composite surfaces was evaluated by roughness assessment and by µ-EDXRF to analyze of calcium and phosphate content in enamel and silica content in composite. Surface roughness and chemical composition of tooth enamel and composite resin were affected by treatments; thus, research hypothesis was accepted.

Tooth bleaching using 38% HP increased the surface roughness of bovine enamel, corroborating with other studies that also showed morphological changes on enamel surface after bleaching treatment (1-4,8,18). On the other hand, previous investigations reported no change on enamel surface (19,20). Probably, chemical composition of bleaching agents and method used to assess the roughness could influence the different results obtained.

Bleaching occurs by HP dissociation in free radicals that penetrate into tooth structure and eventually can reach in the pulp. These free radicals are highly unstable and have capacity to oxidize the chromophores, which are pigmented organic molecules (3,9). After bleaching treatment, as on enamel surface, was also observed increase on roughness of the nanofilled composite, corroborating with findings of other authors (13,14). Organic matrix oxidation of composite by the bleaching agent provides water absorption and loss of the inorganic filler particles, compromising the surface integrity of resinous material (13).

Surface changes on tooth structure and restorative material can appear after toothbrushing, especially when using dentifrices of high abrasiveness (17,21-23), as the whitening dentifrice used in this study. Association of toothbrush bristles with abrasive particles of dentifrice increases the surface roughness of composite by degradation of organic matrix, exposition and loss of inorganic filler during the simulated toothbrushing procedure (21). In this study, increased roughness was observed after simulated toothbrushing for composite resin, as reported by previous studies (16,21), and for tooth enamel (24). On the other hand, the use of singly dentifrice not changes surface roughness of enamel (25). However, toothbrushing procedure associated to bleaching treatment increased the enamel roughness (4,17,22).

In the present investigation, immersion in different low pH beverages increased the bleaching agent and toothbrushing effects on surface roughness of enamel and composite. Acidic drinks consumption can cause deterioration of resinous materials, causing alteration in organic matrix and loss of inorganic filler particles (15). At enamel, acidic beverages promote dissolution of its surface by an erosive process (11). In both cases, these alterations increased the roughness on enamel and composite surfaces, being dependent of exposure time, pH, and chemical composition of solutions (26,27).

In addition, composite resin exposure to ethanol causes decrease on its physical properties (28). Alcohol is a solvent that penetrates the polymer matrix causing surface deterioration and decreases the physical properties of resin-based material (27,29). However, alcohol not causes apparent effects on enamel surface, but the low pH of an alcoholic drink can cause erosive process on this surface (26). Overall, orange juice storage caused higher roughness values for composite and enamel compared to whiskey. Even with close pH values of these two solutions, the citric acid present in orange juice has been considered an aggressive medium be storage tooth hard tissues and resinous materials (30).

Bleached enamel, immersed in acidic drinks, and toothbrushed showed the highest mineral loss. As observed in present investigation, use of different bleaching agents caused calcium and phosphorus loss, assessed by µ-EDXRF and FT-Raman analysis (12). However, no mineral loss after dental bleaching was reported by others studies (5,18). Severity of changes on enamel during bleaching treatment depends of exposure time, concentration, and pH of bleaching agent (1,2,5,8,9), which could explain the controversial results.

The µ-EDXRF analysis of composite resin showed that all treatments promoted silica loss. As for enamel, association of bleaching treatment, immersion in low pH, and simulated toothbrushing caused the highest reduction of silica content. So, oxidation of organic matrix caused by bleaching increases water absorption (13), which combined to abrasion after toothbrushing compromises the composite surface even more, causing loss of filler particles (21). In addition, acidic solutions used also promoted surface erosion as well as the softening of resinous matrix (15).

## Conclusions

Toothbrushing and bleaching association strengthened the effects of superficial changes and chemical composition of tooth enamel as well as composite. Bleaching combined to low pH beverages and brushing caused higher alteration on chemical composition and roughness of enamel and composite.
